# Toward Complete Structured Information Extraction from Radiology Reports Using Machine Learning

**DOI:** 10.1007/s10278-019-00234-y

**Published:** 2019-06-19

**Authors:** Jackson M. Steinkamp, Charles Chambers, Darco Lalevic, Hanna M. Zafar, Tessa S. Cook

**Affiliations:** 1 Department of Radiology, Hospital of the University of Pennsylvania, Philadelphia, PA 19104 USA; 2 Boston University School of Medicine, Boston, MA 02119 USA

**Keywords:** Machine learning, Radiology reports, Natural language processing, Structured reporting

## Abstract

Unstructured and semi-structured radiology reports represent an underutilized trove of information for machine learning (ML)-based clinical informatics applications, including abnormality tracking systems, research cohort identification, point-of-care summarization, semi-automated report writing, and as a source of weak data labels for training image processing systems. Clinical ML systems must be *interpretable* to ensure user trust. To create interpretable models applicable to all of these tasks, we can build general-purpose systems which extract all relevant human-level assertions or “facts” documented in reports; identifying these facts is an information extraction (IE) task. Previous IE work in radiology has focused on a limited set of information, and extracts isolated *entities* (i.e., single words such as “lesion” or “cyst”) rather than complete *facts,* which require the linking of multiple entities and modifiers. Here, we develop a prototype system to extract *all* useful information in abdominopelvic radiology reports (findings, recommendations, clinical history, procedures, imaging indications and limitations, etc.), in the form of complete, contextualized facts. We construct an information schema to capture the bulk of information in reports, develop real-time ML models to extract this information, and demonstrate the feasibility and performance of the system.

## Introduction

Unstructured and semi-structured radiology reports represent an underutilized trove of information for clinical informatics applications, including follow-up tracking systems, intelligent chart search, point-of-care summarization, semi-automated report writing, research cohort identification, and as a source of weak data labels for training image processing systems. The growing importance of structured data is reflected in radiologists’ increasing embrace of structured reporting, standardized coding systems, ontologies, and common data elements [1, 2]. It is an open question as to whether all potentially useful structured information can be captured feasibly a priori at the time of reporting. Even if this undertaking proves feasible, large amounts of historically useful data will remain in their unstructured form.

A parallel approach to procuring structured information involves the development of automated systems capable of extracting structured information from unstructured free text. This approach is frequently used for individual downstream applications (e.g., text mining for research cohort identification). However, individual applications often recreate old pipelines to extract the information from reports, reduplicating large amounts of work, and leaving the general information extraction problem unsolved. Recent advances in natural language processing (NLP) and modern machine learning (ML) technologies have made it feasible to extract complete sets of structured information from unstructured free text within limited domains, essentially fully converting unstructured information to structured information. Tasks of this form fall under the general umbrella of information extraction (IE) [3]. Table 1 provides a breakdown of some (but by no means all) common subtasks and task formulations of information extraction, while Table 2 contrasts rule-based and machine learning approaches for performing these tasks.

**Table 1:** Some of the most common information extraction subtasks

Task formulation	Description	Example	Notes and limitations
Named entity recognition	System provides labels for spans of text within the document, using a pre-specified set of labels. Commonly used for nouns (e.g., radiologic findings and anatomic organs).	Input: “Patient has cyst in the kidney.” Output: Cyst → radiologic finding Kidney → anatomic location	-Does not inherently handle “implied” entities which are not directly mentioned in the text. -By itself, does not link entities to each other, limiting the scope of answerable questions to “which entities appear in this document.” -Traditionally, output is limited to pre-specified entity types.
Relation extraction	In addition to identifying entities, system identifies relations or predicates between entities. For instance, a Finding Is LocatedIn relation may connect a radiologic finding entity with an anatomic location entity.	Input: “Patient has cyst in kidney.” Valid outputs: “Finding Is Located In (cyst,kidney)” / cyst; is in; kidney.	-Can be either “open” (able to identify arbitrary relations between entities) or “closed” (limited to a set of pre-specified relations). -Systems may be limited to binary relations (between two entities) or may handle relations with arbitrary numbers of entities.
Natural language question answering	System provides arbitrary natural language answer to an arbitrary natural language question about a document. System may generate new natural language and/or “point” to spans of text within the document.	Input: “Does the patient have any kidney findings?“ Valid output: “A cyst.” Input: “In what organ is the cyst?” Valid output: “In the kidney.”	-Natural language questions can be seen as a generalization of other natural language tasks (e.g., named entity recognition and relation extraction can both be framed as question-answering tasks). -Natural language answers allow for maximum flexibility compared with a set of pre-specified labels and can ideally generalize to questions outside of the training set. -Requires very large amount of training data, as the task is inherently more complicated. -Providing a complete set of labels for a given training example is difficult, as there may be many correct ways to answer a question.

**Table 2:** Two major approaches for handling information extraction tasks

Methodology	Description	Pros and cons
Rule-based string matching	System uses a human-provided list of rules with particular text strings (or regular expressions) to identify entities and relations within the text.	-Explainable and interpretable. -Simple to implement; may be sufficient for certain limited tasks. -Many natural language words have multiple meanings or classifications based on surrounding context, so it may be impossible to create a true one-to-one mapping between text and labels. -For more complex tasks such as relation extraction and question answering, it is very difficult or impossible to anticipate all rules necessary for the task. -No ability to generalize beyond provided rules.
Machine learning systems	Many available models, e.g., support vector machines and neural networks. System learns from a training data set of input/output pairs and can generalize to unseen examples which are similar. Deep learning models utilizing neural networks are currently state-of-the-art for the majority of complex tasks.	-Many models incorporate information from the entire surrounding sequence of text to produce an answer. -Able to generalize beyond provided training examples. -May be difficult to understand why model produces its output. -Require large amounts of labeled training data.

Previous IE work within radiology has focused on named entity recognition (NER)—identifying particular spans of text corresponding to relevant entities in the document [4–7]. Some definitions of named entity recognition are limited to proper nouns (e.g., person, organization, and location), but in this paper, we use “named entity recognition” to refer to recognition of any text span which corresponds to a relevant piece of information (e.g., including modifiers such as “spiculated” or “5 mm”). Such NER systems represent a step-up from systems which merely perform a raw text search over a pre-specified set of terms, as there are many cases in which there is not a one-to-one mapping between raw text and entity (e.g., homonyms, multiple context-dependent meanings of a word, and pronouns). Furthermore, modern machine learning systems such as neural networks are capable of generalizing beyond set lists of terms and are therefore not limited by incomplete ontologies.

However, systems which strictly perform named entity recognition-level tasks are insufficient for answering even basic clinical queries. Perhaps the most commonly cited example is negation: in the sentence “No lesion observed,” an NER-only system could (correctly) identify “lesion” as an entity, but cannot correctly answer the intended question “does this patient have a lesion” without additional information about the negation and how it relates to the “lesion” entity. Many other limitations exist, including conditional expressions, e.g., “if a lesion is present, consider …”; uncertainty, e.g., “possibly representing a lesion”; temporal modifiers, e.g., “patient with history of lesion”; and even person-specific, e.g., “patient’s father with reported history of lesion”; which add crucial context to the entity. Despite the relatively narrow vocabulary and informational scope of radiology reports compared with general-purpose English text, these linguistic phenomena are very common in radiology reports, rendering pure NER systems incapable of answering basic clinical queries without additional post-processing.

The task known as relation extraction [8] is a step-up from NER (see Table 1), as it involves identifying entities *and* relations between them. In the most general form, a set of possible relations between entity types are defined, such as “lesion X has size Y” or “imaging modality X was used on body region Y”, and a successful system is capable of identifying these relations from raw text. Such systems, therefore, *can* answer questions such as “does this patient have a lesion,” “are any of the lesions larger than 5 mm,” or “has this lesion changed since it was last observed” by using information *relating* multiple named entities. Some systems exist for relation extraction in a general-purpose clinical text, but the radiology literature is limited.

In addition, most systems only focus on small subsets of information within reports. By contrast, we aim to create a system capable of general-purpose complete information extraction from radiology reports, for a wide variety of downstream uses, as listed above. In this study, we develop an information schema capable of capturing the majority of information in radiologic reports, demonstrate the feasibility of a neural network system to extract this information, and motivate possible downstream uses.

## Methods

### Fact Schema

We iteratively developed a schema of all relevant information documented in radiology reports by qualitatively examining a body of existing reports. For the purposes of this feasibility study, we used only abdominal and pelvic radiology reports to constrain the space of possible information. However, most of the schema covers information (e.g., findings, follow-up recommendations, and indications) which applies to all body parts, and a similar process could easily be used to modify the informational schema to include missing domain-specific information.

**Fig. 1:**
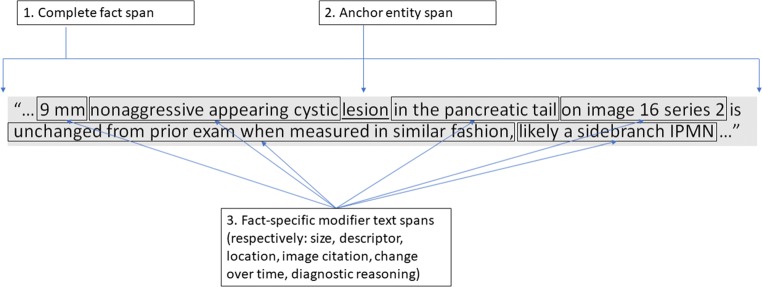
Example fact with anchor and modifier text spans

The basic unit of information in our schema is a clinical assertion, predicate, or “fact”, such as “a radiologic finding was observed,” “the patient has a follow-up recommendation,” or “the patient has a known diagnosis.” Each fact has one “anchor” text span—e.g., the finding, the recommendation, or the diagnosis—as well as a set of informational “modifier” text spans which contextualize or modify the fact. These modifier spans include common linguistic elements such as negation, uncertainty, and conditionality, as well as fact-specific information (e.g., a radiologic finding has “location,” “size,” “description,” and “change over time” whereas a follow-up recommendation has “desired timing”) designed to be able to answer most reasonable queries a clinician might have regarding a report. These modifier spans are roughly analogous to “slots” in a *slot filling task* [9], in which systems populate predefined relation types for a given entity. Figure 1 shows an example “radiologic finding was observed” fact.

It is important to note that multiple facts can occur within the same span of text—for instance, a sentence stating “this exam is compared to a previous abdominal CT” contains at least two separate facts—(1) that an imaging study was done in the past and (2) that this imaging study was used for comparison with the current study.

Rather than trying to build up an ontology from simple entities, as past work has done, we designed each anchor and modifier span to answer a specific question and trained our system to produce all information necessary to answer the question implied by the span type. For instance, in a “patient has radiologic finding” fact, the entire phrase “within the left kidney” would be tagged as the “location” span, as all of this information, including the organ, laterality, and preposition “within,” is necessary to answer the common question “where is the finding?”

We iteratively developed the information schema by using it to label radiology reports with their complete factual content. When information was found that was not captured by our schema, we updated our schema to capture this information. The schema is extensible to any piece of information which can be formatted as assertions or predicates relating groups of entities which map to spans of radiologic text.

### Data

For our data set, we used a convenience sample of 120 abdominal and pelvic radiology reports from imaging studies obtained at our institution from 2013 to 2018. These reports included a variety of indications as well as modalities, including CT (*n* = 50), MRI (*n* = 48), and ultrasound (*n* = 22). The reports included adult patients of all ages and sexes. The reports were written using a wide variety of personal reporting templates and language and most were written in prose. Some reports, but not all, used some form of anatomical section headings, although the form of these headings was highly variable. The corpus included reports written by 26 different attending radiologists and 51 resident radiologists. Our study was approved by our Institutional Review Board.

We used custom-developed labeling software to manually annotate 120 reports with their complete factual content. Each piece of extracted information was required to be linked to a specific span of text in the document, in order for the system to be interpretable and evaluable. Each fact was labeled with the following three pieces of information: (1) the minimal contiguous text span from the report necessary to capture the complete fact, (2) the text span corresponding to the anchor span which defines that fact, and (3) text spans corresponding to surrounding pre-defined modifier spans (e.g., negation and size of lesion). Labels [2] and [3] were required to fall within the span of label [1], but only the anchor entity [2] was required to be contiguous. A fact could have only one anchor entity, but could have any number of non-anchor modifier spans, including zero. In many cases, the same text span was labeled with multiple different fact instances. Text spans for all three span labels were constrained to the word token level rather than the individual character level (e.g., a fact might begin at token 230 and end at token 266). Tokenization of documents was performed using spaCy, a freely available python package.

### Models

For continuous word token embeddings, we used custom fastText vectors trained on a corpus of 100,000 abdominopelvic radiology reports at our institution. We chose fastText because it is relatively quick to train and is capable of using subword information such as a word’s spelling, enabling out-of-vocabulary tokens (including typos) to be reasonably well-represented. We used the fastText implementation from the gensim python package with the skip-gram training procedure, 300-dimensional embeddings, and all other parameters set to gensim’s default values. Qualitative inspection of the resulting vectors showed reasonable similarity between nearby words both in spelling and semantics. We concatenated these embeddings with GloVe embeddings trained on large volumes of general-purpose English text to form our complete word embeddings, believing that the combination of large-volume training data (from GloVe) and domain-specific embeddings (from fastText) may outperform each embedding type individually.

We used a two-part neural network architecture for our models. The first neural network takes a full document as input and outputs (1) token-level predictions of anchor entities and (2) token-level predictions of complete fact spans. The second takes as input an anchor entity, fact type, and candidate fact span and outputs (1) token-level predictions for context modifier spans and (2) a refined prediction of the beginning and end of the complete fact. This refinement is necessary because of the small amount of training data for the full-document model (120 example documents) compared with the large amount of training data for the second network (> 5000 manually labeled fact spans within these reports). It is unsurprising that the fact spans predicted by the fine-tuning module in the second neural network are significantly better than those predicted by the first neural network, given the significantly larger volume of training examples it had to work with.

The first neural network works as follows: embedded full documents are processed sequentially by a 2-layer bidirectional gated recurrent unit (GRU), a type of recurrent neural network module used frequently in text processing systems to encode surrounding context. The outputs of the two directions of the GRU are concatenated and used as shared features for two separate dense layer “heads,” which calculate predictions for each word token in the document. One head predicts which fact types each word token is part of and the other predicts which anchor entity types (including none) a word token is part of. We treated the problem as a multi-class, multi-label prediction problem, allowing each word token to be part of multiple different facts or anchor types. A dropout of 0.3 was applied to the GRU outputs for regularization. We opted to keep the fact span head and anchor span head independent, and use the joint output of both to definitively identify facts, as described below.

For every predicted anchor span, if it is inside of a predicted fact span, we create a fact candidate. The second neural network takes as input each fact candidate, consisting of the predicted anchor span, the predicted fact type, and the embeddings of the words within the predicted fact span. The predicted anchor span is provided in the form of a mask vector of zeros and ones, where 1 corresponds to a predicted anchor token and 0 corresponds to a predicted non-anchor token. This “mask” vector is concatenated to the word embeddings. To enable the second network to expand the predicted span of the first network if necessary, we also include the word embeddings of the 20 tokens which come before and after the predicted fact span. This network has separate weight parameters stored for each separate fact type, as each fact may require different learned information—the fact type predicted by the first network determines which stored parameters are used by the second network.

The second network outputs word token-level predictions for all modifier spans (including possible refinement of the anchor entity prediction), and error is computed using a sigmoid function followed by binary cross-entropy loss. Last, this network predicts the refined beginning and end of the complete fact span as follows: a dense layer processes the GRU-encoded tokens and outputs “beginning” and “end” scores for each token. A maximum is taken over all token positions to produce the final predictions for the beginning and end of the fact span, and loss is computed using a softmax followed by a cross-entropy loss function. The overall loss function for the second network is the sum of the beginning and end position losses and the token-level predictions.

**Fig. 2:**
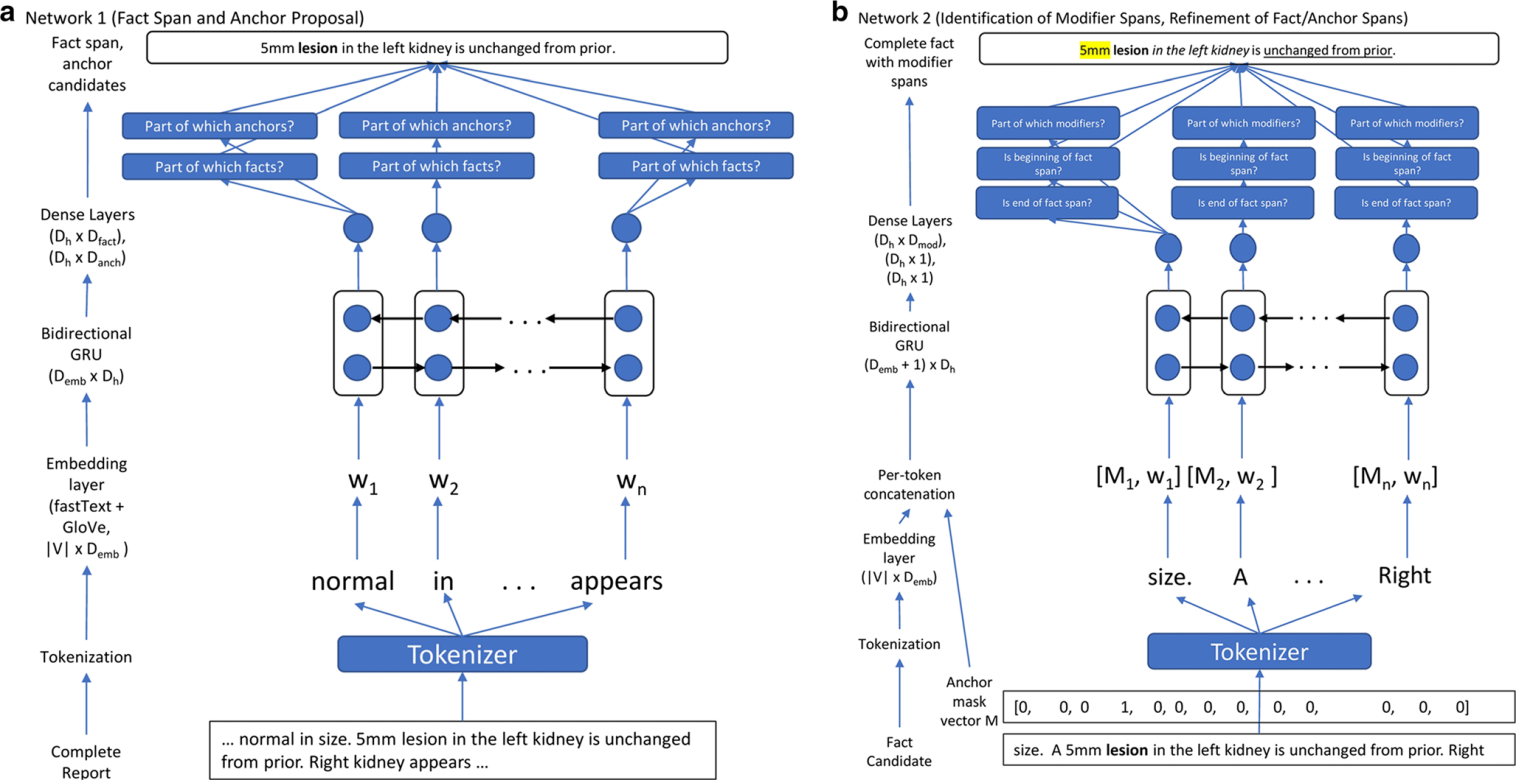
Architecture for (**a**) first and (**b**) second neural network

A complete diagram of the neural network architecture is given in Fig. 2.

### Training and Validation

Data was divided into training, validation, and test sets (80%/10%/10%) and model design decisions and hyperparameters were tuned on the validation set. Models were trained until validation set performance ceased increasing, with a patience of three epochs. Model performance was evaluated using word-level micro-averaged recall, precision, and F1 score for (1) full-fact spans, (2) anchor entities, and (3) modifier spans on the unseen test data.

For further information about model hyperparameters and other specific training decisions, see Appendix.

## Results

### Information Schema

Our full information schema is given in Table 3, along with parsed examples and relative frequency in the labeled document corpus. In total, 5294 facts were labeled (an average of 44.1 facts per report) with 15,299 total labeled pieces of information (anchor entities and modifier spans) for an average of 2.88 labeled text spans per fact. Our fact schema was comprehensive and covered the vast majority of information present in abdominopelvic radiology reports, with 86.3% of the raw text (53,862 tokens) covered by at least one fact. Almost all of the texts not covered by facts were document section headers and other metadata. We did not have to add any new facts after approximately 50 reports were fully labeled, suggesting that we had achieved some degree of content saturation within the limited domain of abdominopelvic reports.

**Table 3:**
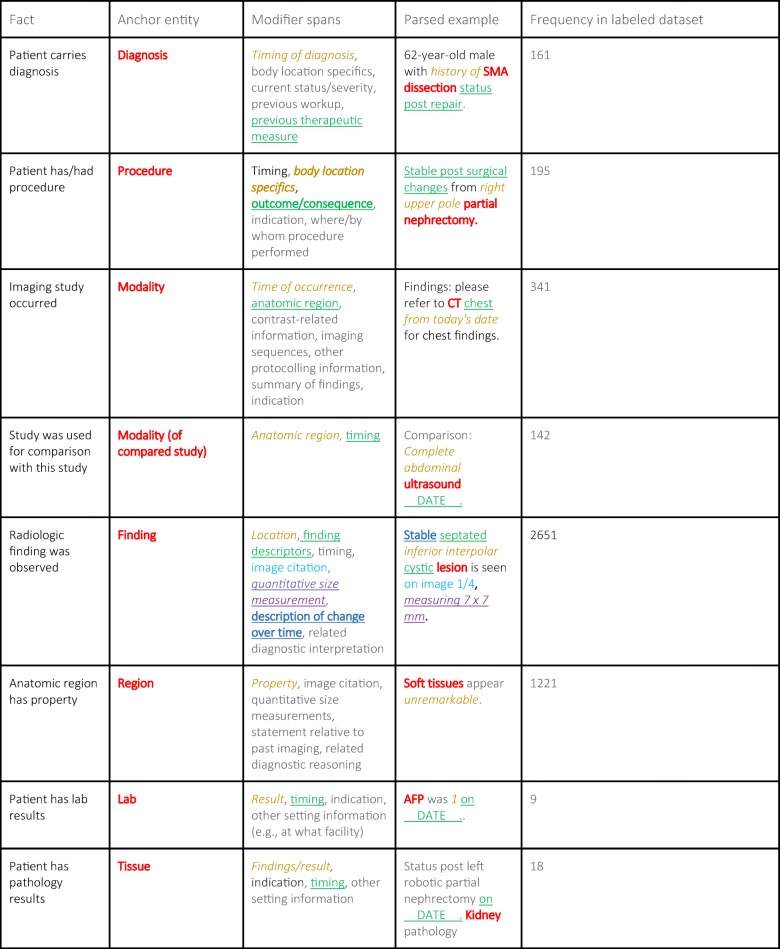

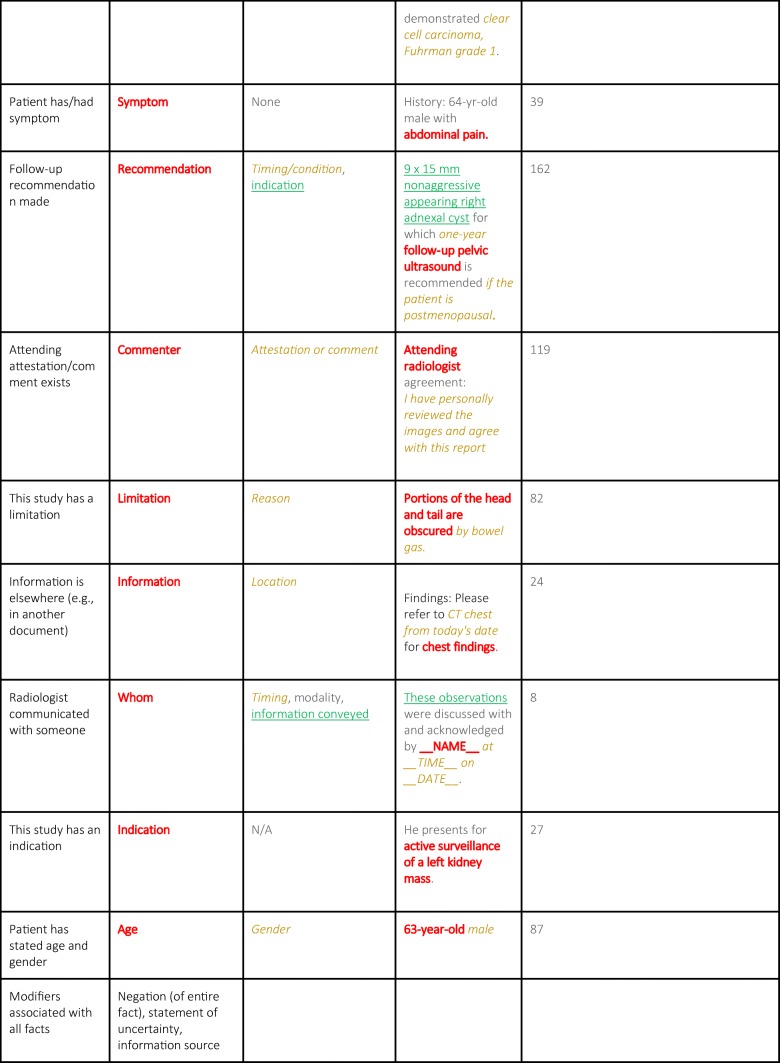
Complete information schema. Examples are color-coded using the colors in the “anchor entity” and “modifier spans” columns. Modifier spans which are in standard color do not appear in the parsed example, while example texts in standard color are not part of any information spans. Note that multiple modifier and/or anchor spans may overlap, but for ease of visualization, no examples with overlapping spans were selected

### Fact Extraction

For prediction of fact spans, our model achieves token-level recall of 88.6%, precision of 93.5%, and F1 of 91.0%, micro-averaged over all fact types. For anchor prediction, we achieve 77.1% recall, 88.4% precision, and 82.4% F1. Micro-averaged across all pieces of information, including modifier spans, the model achieves 74.5% recall, 75.1% precision, and 74.8% F1. Performance varies significantly across class types, from 0% for rare facts (i.e., < 10 training examples) to > 95% for the most common facts. This is unsurprising given that some fact types have extremely limited training data in our corpus; e.g., in our corpus, there were only 9 “patient has lab result” facts vs. approximately 2500 “radiologist asserts imaging finding” facts, so the model does not have enough data to learn generalizable properties for the rarest fact types. Additional data would likely improve the model’s performance across all fact types, but most particularly on rare fact types. However, our model was able to achieve > 75% F1 score at extracting “patient had procedure” and “patient carries diagnosis” facts, each of which have fewer than 200 labeled training examples, suggesting that the volume of training data required for this model to perform well in limited domains is not prohibitively large. Future labeling can be directed toward the rare facts to improve the system’s performance.

### Examples

Inspection of predictions on unseen reports showed that the network was able to extract varied information successfully from complex sentences. For instance, in the test set fact “9 mm nonaggressive appearing cystic lesion in the pancreatic tail on image 16 series 2 is unchanged from prior exam when measured in similar fashion, likely a sidebranch IPMN,” the system was able to produce correct labels for all 7 informational spans, including diagnostic reasoning. Furthermore, the system was able to generalize to fact instances it had not seen before (for instance, it identified “Gamna-Gandy bodies” as a finding in the test set), indicating that it had learned general representations of how facts are recorded in radiology reports. In this way, it has a significant advantage over rule-based systems and ontologies which are limited to pre-specified vocabularies and ontologies. The fastText embeddings, which utilize subword information in the form of character *n*-grams to embed unseen words, allowed the system to handle typographical errors effectively.

## Discussion

Our study demonstrates the feasibility of near-complete information extraction from radiologic texts using only a small corpus of 120 abdominopelvic cross-sectional imaging reports. It introduces a novel information schema which is capable of handling the vast majority of information in radiologic text and a neural network model for extracting that information. It is capable of extracting information from reports written in a wide variety of template and prose styles by > 50 different radiologists. Although we have not yet integrated it into the clinical workflow, the system is capable of operating in real time (i.e., while a user types) and the information schema is easily extensible to additional facts with no changes to the neural network other than the number of output units. It is likely that our schema and models would generalize effectively to other radiologic and clinical domains.

Our representational system has several strengths. First, it is capable of handling multiple facts of the same type contained with the same text span—for instance, in the sentence “hepatic artery, portal vein, and hepatic veins are patent,” our system extracts three separate “anatomic region has property” facts - one for each anatomic structure. This disentanglement of separate facts from raw text is particularly crucial for downstream applications that involve tracking or reasoning over discrete facts. Second, by representing our information schema using complete *predicates*, e.g., “patient has diagnosis” and “imaging study was used for comparison with this study,” we are able to represent relations involving some implied entities such as “this patient” or “this study” and differentiate them from predicates involving other people or studies. Thirdly, by treating our modifier spans as *questions to be answered* rather than *entities to be identified*, we include all the information necessary to answer a single clinical query in each modifier span. To see the advantage of this, consider the sentence “Lesion observed within the left kidney.” Many NER-based systems would attempt to identify “left” as a laterality and “kidney” as a body organ, but our system identifies the complete text span “within the left kidney” as the location of the lesion, which directly answers the clinical query and is more readily applicable to downstream tasks; e.g., an automated tracking system could disambiguate this lesion from other kidney lesions or lesions on the left side of the body, and display it separately.

There are, however, some limitations to our representation of the IE task. Although our system is able to represent the vast majority of information found in abdominopelvic radiology reports, no matter how comprehensive a structured information schema is, there are rare types of information that will be missed. Furthermore, our system is unable to handle some types of “implied” information which are not directly referenced in the text–e.g., in the sentence “The gallbladder is surgically absent,” an ideal system might infer a cholecystectomy procedure fact, but there is no span of text corresponding to the procedure name in this sentence, and therefore our system cannot extract a procedure fact. For this study, this was a deliberate design decision, as we wanted to provide interpretable and verifiable output where each prediction is linked to a particular span of text [13]. However, this trade-off results in our system being unable to extract some types of *implied* information. In the limited domain of radiological text at our institution, the assumption of direct textual evidence for each entity was valid in the vast majority of cases, but not all.

Exceptions are rare in the structured domain of radiologic text, and we were able to find sensible workarounds for this study. However, moving to a more general model, where systems are capable of answering *arbitrary natural language questions* about a report or span of text, is likely necessary to solve some of these difficulties and expand the capabilities of information extraction systems. Figure 1 of a study from the Allen Institute [10] effectively highlights the difficulty in capturing every piece of information using structured ontologies and relations between entities and suggests a simpler alternative to complex ontologies in the form of *question-answer meaning representations (QAMRs)*, which do not grow more complex and brittle with larger information schemata. Development of large question-answer data sets for radiologic and other clinical domains is an important step for progressing the field and developing models which can effectively comprehend and reason about radiologic texts. A parallel approach involves transfer learning from general-purpose question answering tasks such as the Stanford Question Answering Dataset (SQuAD) [11].

Another limitation of this study is the small amount of labeled data available for the full document-level neural network (120 reports); it is a general rule that deep learning models perform better with larger training data sets, and many specific vocabulary terms are not represented within this corpus. Labeling each report with their complete informational content requires hundreds of text spans to be manually identified (each report takes 30–45 min to label). However, our models were able to perform reasonably well even with this small training set and are able to generalize unseen vocabulary terms, suggesting that the effort required to create effective training sets is not prohibitive even for smaller groups of clinicians or researchers. We plan to continue increasing the size of the training set, including additional reports from other department sections, imaging modalities, and institutions, to improve generalization performance. Alternatively, using rule-based labeling algorithms to produce weak labels for a much larger corpus of reports may be effective for training, although manually labeled data is still required to evaluate the performance of such systems.

From the modeling perspective, usage of more sophisticated word token representations such as language models, which tend to outperform static word embeddings on most NLP tasks [12] may provide further improvement. Using attentional models such as the transformer architecture [13] may also enable the system to handle long-range text dependencies more effectively than recurrent neural network models.

Our current study is limited to abdominopelvic radiology reports, and we did not conduct any formal experiments on other report types. It is likely that the similar vocabulary describing findings, recommendations, imaging modalities, and measurements, as well as the similar document-level form of most reports (findings ordered by anatomic region) would provide a baseline level of generalizability across radiology subdisciplines. However, exposure to subdiscipline-specific vocabulary (anatomic terms, specific lesion descriptions) would almost certainly be necessary for high-accuracy performance. Future work will aim to formally test these assumptions (e.g., training a model on one type of report and testing on another).

## Conclusions

We develop a comprehensive information schema for complete information extraction from abdominopelvic radiologic reports, develop neural network models to extract this information, and demonstrate their feasibility. Our system uses no pre-specified rules, runs in real time, generalizes arbitrarily large information schemata, and has representational advantages over systems which only perform named entity recognition. The system has many downstream applications including follow-up tracking systems, research cohort identification, intelligent chart search, automatic summarization, and creation of high-accuracy weak labels for large imaging data sets. Future work includes using more sophisticated language models, generalizing to all radiologic subdisciplines, and building downstream applications. Complete conversion of unstructured data to structured data represents a feasible complementary approach to structured reporting toward the goal of creating fully machine-readable radiology reports.

## Appendix. Hyperparameters and Model Specifics

All models were trained using the PyTorch module with the adaptive moment estimation (Adam) optimizer and a learning rate of 1e-3.

Since the second network receives as input the predicted fact spans and anchors from the first network, we stochastically augment the dataset to simulate incorrect output predictions by the first network. A random number of additional tokens between 0 and 20 is appended to either side of the true fact span in order generate a noisy training example for the second network. This enables the second network to learn to correct the first network’s incorrect predictions.

Training was completed in 2 h on a machine with one NVIDIA GTX 1070 GPU.
